# Type 1–3 Canal Configuration in the Buccal Root of a Maxillary Second Molar

**DOI:** 10.1155/2022/8991579

**Published:** 2022-09-01

**Authors:** Hui Li, Qiang Wang, Mingwen Fan, Qingan Xu

**Affiliations:** ^1^Department of Operative Dentistry and Endodontics, Wuhan First Stomatological Hospital, No. 675 Jianshe Avenue, Jianghan District, Wuhan 430000, China; ^2^Department of Stomatology, School of Medicine, Jianghan University, No. 8 SanJiao Lake Road, Economic and Technological Development District, Wuhan 430000, China

## Abstract

**Objectives:**

A major challenge in root canal treatment is the complex and variable root canal system. There are several methods of classification of root canal systems. Herein, we report a case of a maxillary second molar, the root canal system of which could not be classified according to any of the previous methods. *Case Report*. Cone-beam computed tomography (CBCT), used to visualize the root canal system of a maxillary left second molar with fused roots, revealed a type 1–3 root canal system in the buccal root and a type 1–1 root canal system in the palatal root. A dental operating microscope was used throughout the treatment, and the root canals were successfully obturated.

**Conclusion:**

To the best of our knowledge, this is the first report on the classification of buccal roots of maxillary second molars. *Clinical Significance*. A dental operating microscope and CBCT play a vital role in mapping the root canal system to ensure that no canal is missed.

## 1. Introduction

A major challenge in root canal therapy is the existence of complex and variable root canal systems. To fully understand the internal morphological changes in different teeth, several studies have been conducted, and root canal systems have been classified into different categories [1, 2]. This provides the dentist with information about the root canal system. When encountering root canal systems beyond the classification, the dental operating microscope (DOM) is an effective device to help dentists identify specific anatomic features of the root canal [3]. The minimum distance that the human eye can distinguish is 0.1 mm, and more details can be visualized using a DOM. Moreover, use of a DOM ensures that the ultrasonic tip accurately removes the calcification that blocks the root canal orifice, reduces unnecessary grinding of healthy dentin, and improves the fracture resistance of the teeth [4]. In addition, cone-beam computed tomography (CBCT) can predict root canal location in teeth with unusual root canal anatomy [5]. Vertucci described three types of root canal systems in maxillary second molars. The variation mainly occurs in the mesiobuccal root, which is divided into type I, type II, and type IV [1]. Regarding the maxillary second molar, some studies have reported three canals in the mesiobuccal root [6], while other studies have reported two canals in the palatal root [7–10]. Herein, we present a case of a maxillary left second molar with a trifurcated buccal root canal. The DOM and CBCT showed two canals at the orifice level; however, the buccal canal trifurcated at the middle third of the root. To the best of our knowledge, there is no relevant report on the classification of buccal roots of the maxillary second molar.

## 2. Case Presentation

**Figure 1:**
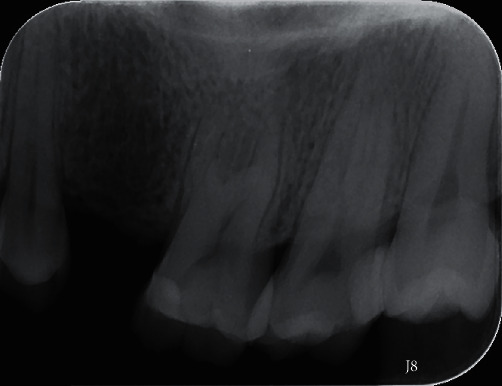
Preoperative radiograph.

A 44-year-old woman presented to the Department of Operative Dentistry and Endodontics with pain, both spontaneous and to extreme temperatures, on the left side of the face, two days prior to her visit. Clinical examination revealed dental restoration in teeth #15, which had been placed one year prior at a different hospital. Tooth #15 had severe pain on percussion, and a severe lingering painful response to cold water, whereas tooth #14 was only tender to percussion. The periodontal conditions of teeth #14 and #15 were normal. Tooth #15 was diagnosed with irreversible pulpitis. The patient was informed about the conditions of her teeth and was advised to undergo root canal treatment (RCT) on tooth #15. The patient provided informed consent for RCT. The preoperative radiograph (Figure 1) showed dental restoration approximating the pulp chamber in tooth #15.

**Figure 2:**
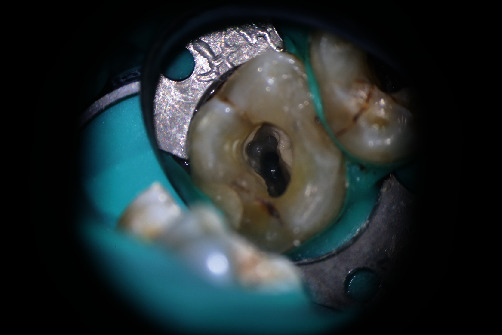
Three canals in the buccal root.

**Figure 3:**
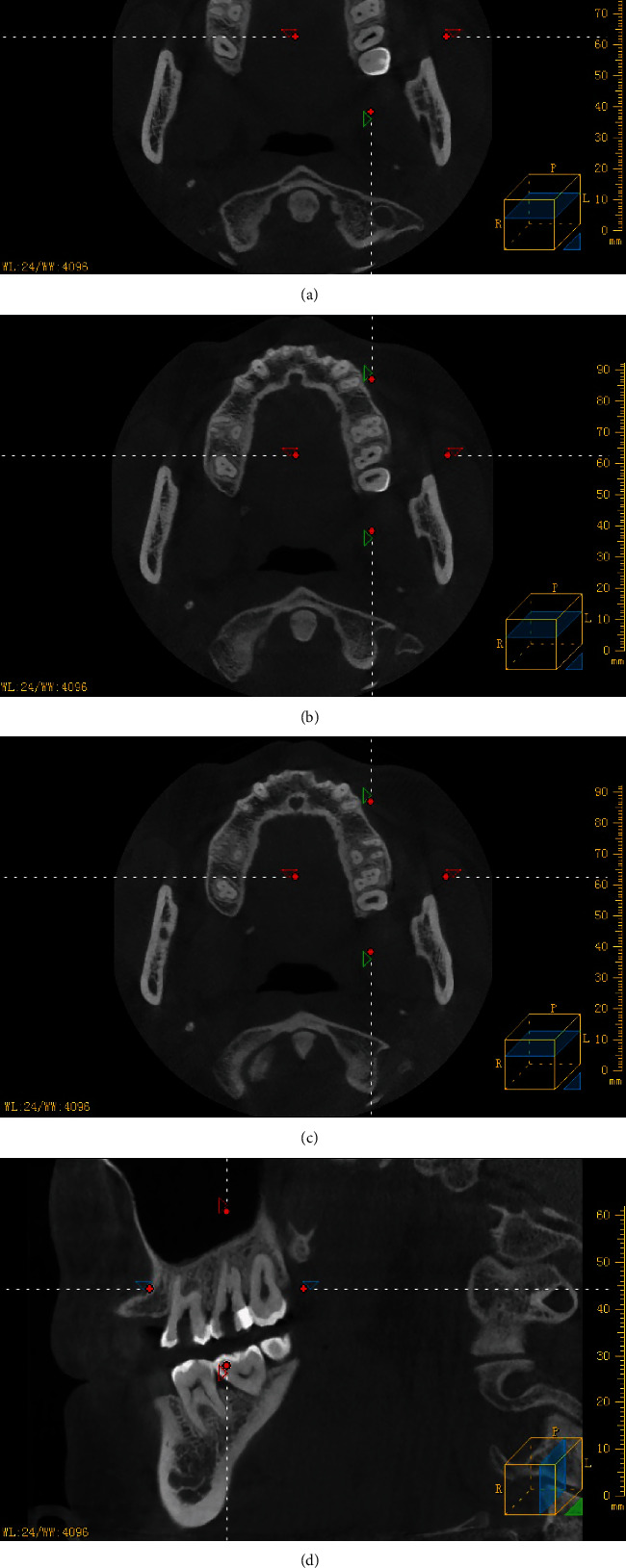
Cone-beam computed tomography images. Horizontal sections at (a) orifice level, (b) midroot level, (c) apical level, and (d) sagittal view reveal a type 1–3 canal.

After administration of local anesthesia, an access cavity was prepared in tooth #15 and paraformaldehyde was placed into the pulp chamber to inactivate the dental pulp. Seven days later, the temporary seal was removed, and the root canals were visualized under a DOM (Zumax, Suzhou, China). A #10 K file (Dentsply Sirona, York, PA) was used to explore the root canals under 5× magnification using the DOM. The resistance to exploration was significantly lesser in the buccal root canal than in the palatal root canal. Next, the magnification was set to 20×, and a C-shaped root canal was observed in the buccal root with some dentin blocking the direct access. An ultrasonic working tip (ET20, Satelec, France) was used to remove part of the dentin; four root canals were found, three of which were in the buccal root and had a common orifice, and one was in the palatal root (Figure 2). The patient was recommended to undergo CBCT (NewTom, Italy) because of the morphological variation in tooth #15 to avoid missing other canals. CBCT showed that the root canal was trifurcated at the middle third of the fused buccal root, and the palatal root contained a single independent root canal (Figure 3). An electronic apex locator (Propex II, Dentsply, USA) was used to measure the length of the root canal, and pathfiles #13, #16, and #19 (Dentsply Sirona, York, PA) were used sequentially to dredge the root canals. The canals were prepared using #20 and #25 Protaper-next nickel–titanium rotary instruments (Dentsply Sirona, York, PA). Ethylenediaminetetraacetic acid (EDTA; Glyde, Dentsply, USA) was used for each root canal preparation. Frequent irrigation was performed using 2.5% NaOCl with P5 Newtron ultrasonic agitation (Satelec, France). The canals were rinsed, dried, and filled with calcium hydroxide paste. The access opening was sealed with a zinc oxide and eugenol dressing.

**Figure 4:**
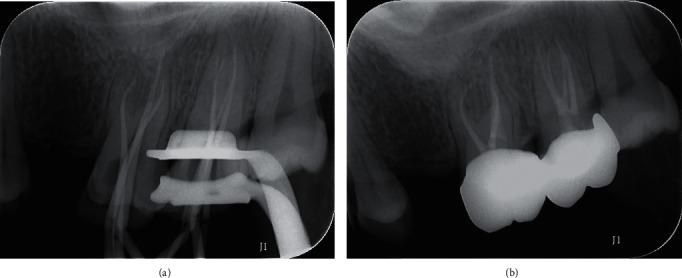
(a) Radiograph to confirm the working length, (b) postoperative radiograph.

Seven days later, the canals were emptied and copiously flushed with 2.5% NaOCl. Ultrasonic irrigation of the root canals was performed, followed by drying with paper points. Master gutta-percha cones were selected for all canals (Figure 4a), and the canals were obturated using iRoot SP (Innovative BioCreamix, Vancouver, Canada), using the single-cone technique. Two weeks later, a computer-aided design/manufacturing ceramic crown was fabricated on tooth #15. A final radiograph (Figure 4b) was taken to confirm the completeness and extension of the root filling.

## 3. Discussion

Despite the high success rate achieved in RCT, morphological variations in the root canal are an unresolved mystery. Variations in root canal anatomy are closely associated with failure of RCT because of the difficulty in locating, cleaning, and filling the aberrant canals [1, 2, 6–12]. Owing to the many dissimilarities in selection, the results of previous studies cannot be compared directly. In an *in vitro* study, 42.25% of 187 extracted maxillary second molars had fused roots. Among teeth with fused roots, those with three-root fusion had a high frequency of merged canals [10]. Another study using CBCT to evaluate the incidence of root fusion and root-canal fusion in 4120 molars *in vivo* found that a complex root canal system is often present in fused roots. In this study, the incidence of root fusion and root-canal fusion in the maxillary second molar was 25.2% and 8.6%, respectively [13]. Another *in vivo* study using CBCT showed a 23.9% incidence of fused roots in maxillary second molars, with root-canal fusion within fused roots observed in 10.6% of cases [11]. In our patient, CBCT showed a three-root fusion in tooth #15. Ordinola-Zapata et al. reported that maxillary second molars with fused roots have a high incidence of merged and C-shaped canals [12].

In our case, tooth #15 showed a distinctive variation. Two separate root canals originated from the pulpal floor: one buccal and one palatal canal. Under the DOM, a C-shapedconfiguration was visualized till the middle third of the buccal root, which trifurcated in the mesial, buccal, and distal directions and formed three independent root canals with independent apical foramina. In this case, the treatment was difficult because we had to locate and clean the three buccal canals and put three gutta-percha cones through the same orifice at the same time. Fortunately, DOMs are being increasingly used for endodontic treatment, and are especially useful in such cases. CBCT can accurately image the maxillofacial hard tissue and confirm the internal anatomy of the teeth that need RCT [14–16].

At present, irrespective of the instruments used, some areas of the root canal cannot be cleaned. Therefore, chemical disinfection of root canals is the key to successful treatment [17]. In our case, EDTA was used to remove the smear layer and NaOCl and ultrasonic irrigation were used to reduce bacterial load and dissolve organic tissues, thereby improving the success rate.

## 4. Conclusion

Finding and treating additional root canals can improve the success rate of RCT. Therefore, clinicians should be aware of the typical root canal configuration. On encountering an aberration, clinicians should ask questions and use the DOM and CBCT in a timely manner to deal with various complex situations. Particularly, during RCT of the maxillary second molar, if a fused root canal is suspected on radiographs, it is recommended that the entire treatment be performed under a DOM. CBCT should be performed to confirm the root shape and number of root canals to avoid missing root canals. We believe that the prognosis of RCT can be improved with standardized treatment combined with the use of advanced equipment.
